# *Mycoplasma pneumoniae*-triggered hemophagocytic lymphohistiocytosis complicated by type I plastic bronchitis in a child: a case report

**DOI:** 10.3389/fimmu.2026.1944516

**Published:** 2026-10-29

**Authors:** Mengyao Zhang, Jiafeng Zheng, Caili Zhou, Yongsheng Xu

**Affiliations:** 1 Department of Pediatric Respiratory Medicine, Children’s Hospital, Tianjin University/Tianjin Children’s Hospital, Tianjin, China; 2 Pediatric Hematology Department, Children’s Hospital, Tianjin University/Tianjin Children’s Hospital, Tianjin, China

**Keywords:** case report, hemophagocytic lymphohistiocytosis, *Mycoplasma pneumoniae*, NK-cell degranulation, plastic bronchitis

## Abstract

**Background:**

Hemophagocytic lymphohistiocytosis (HLH) triggered by *Mycoplasma pneumoniae* (MP) is exceptionally rare in children, with fewer than 25 pediatric cases reported. Its concurrence with plastic bronchitis (PB) has not been well characterized, and host factors predisposing to recurrent infection-triggered hyperinflammation remain poorly understood.

**Case presentation:**

A 6-year-old previously healthy girl experienced two hyperinflammatory episodes separated by 7 months. The first episode (August 2023) featured fever, lymphadenopathy, hepatosplenomegaly, hyperferritinemia (4,184 ng/mL), elevated cytokines, and declining blood counts but did not fulfill complete HLH-2004 criteria; it resolved with hepatoprotective therapy and a short corticosteroid course. The second episode (March 2024), triggered by confirmed M. pneumoniae infection (targeted next-generation sequencing), fulfilled 7 of 8 HLH-2004 criteria: fever, splenomegaly, pancytopenia (hemoglobin nadir 72 g/L, platelets 32 × 10^9^/L, ANC 0.86 × 10^9^/L), hypertriglyceridemia/hypofibrinogenemia (0.748 g/L), hyperferritinemia (31,905 ng/mL), decreased NK-cell activity, and bone marrow hemophagocytosis, with elevated soluble CD25 (9,249 pg/mL, approximately 2,312 U/mL—marginally below the 2,400 U/mL threshold but indicative of significant T-cell activation) and an HScore of 231 (>99% probability). Complications included severe hepatic injury (AST 4,017 U/L), consumptive coagulopathy, toxic–metabolic encephalopathy with seizure, and histologically confirmed type I plastic bronchitis requiring two bronchoscopies for bilateral cast extraction. Immunological evaluation revealed borderline NK-cell degranulation (ΔCD107a 5.68%; severe impairment threshold ≤5%) with normal CTL degranulation and preserved perforin, MUNC13-4, SAP, and XIAP expression. Quantitative NK-cell deficiency had been documented during the first episode. Treatment with modified HLH-2004 protocol (dexamethasone and IVIG without etoposide), doxycycline, and supportive care achieved complete recovery.

**Conclusion:**

This case demonstrates that MP-HLH can present with exceptional severity including type I PB and multiorgan failure, particularly in children with prior hyperinflammatory episodes suggesting underlying immune vulnerability. Borderline NK-cell degranulation combined with quantitative NK-cell deficiency and recurrent hyperinflammation supports a threshold model of HLH susceptibility. Functional immune screening including CD107a degranulation assays should be performed in children with recurrent or unusually severe infection-triggered hyperinflammation, even without a definitive monogenic diagnosis.

## 1 Introduction

Hemophagocytic lymphohistiocytosis (HLH) is a life-threatening hyperinflammatory syndrome driven by uncontrolled activation of cytotoxic T lymphocytes and macrophages, excessive cytokine production, and multiorgan injury (1, 2). Diagnosis requires an HLH-associated molecular abnormality or fulfillment of at least five of eight HLH-2004 criteria (2). Although traditionally classified as primary (germline cytotoxicity defects) or secondary (triggered by infections, malignancies, or autoimmune conditions), this dichotomy increasingly represents a continuum in which environmental triggers interact with host genetic and functional susceptibility (1–3). HLH remains highly lethal, with a pooled mortality of 32.6% in critically ill children (4).

Mycoplasma pneumoniae (MP) is a common respiratory pathogen in school-aged children that can also provoke marked immune dysregulation. MP-associated HLH (MP-HLH) is exceptionally rare; an early series described only four pediatric patients with generally milder cytopenias than EBV-associated HLH (5), and cytokine profiling has suggested distinct immunopathogenic features (6). A recent review identified only 23 pediatric cases, with higher mortality when diagnosis was delayed beyond 2 weeks (7). Because persistent fever, cytopenia, and pulmonary deterioration may be attributed to refractory MP pneumonia, MP-HLH is probably underrecognized. Recurrence after separate infections and host factors predisposing to such recurrence remain poorly characterized.

Plastic bronchitis (PB) is a rare disorder in which branching casts obstruct the tracheobronchial tree, causing atelectasis and respiratory failure. Type I casts contain fibrin and inflammatory cells, whereas type II casts are acellular and mucinous (8). MP is an established cause of type I PB through intense airway inflammation and cast formation (9). Notably, PB was not documented among the 23 pediatric MP-HLH cases in the recent review (7), indicating that their concurrence is exceptionally unusual.

Here, we report a child with recurrent hyperinflammation whose second episode—confirmed MP-triggered HLH—was complicated by type I PB, and examine a subclinical NK-cell degranulation defect as a potential susceptibility factor. CD107a mobilization assays evaluate cytotoxic-granule exocytosis and can identify degranulation abnormalities associated with HLH predisposition (10, 11). This case highlights the value of functional immune assessment in children with recurrent infection-triggered hyperinflammation, even when a definitive monogenic diagnosis is not established.

## 2 Case presentation

### 2.1 First episode (August–September 2023)

A 6-year-old previously healthy girl was admitted to Tianjin Children’s Hospital on August 28, 2023, with a one-month history of polymorphic skin rash, intermittent fever (peak 40 °C) for over 20 days, and asymmetric polyarthralgia involving the wrists, ankles, and proximal interphalangeal joints. Physical examination revealed generalized lymphadenopathy (bilateral cervical, supraclavicular, axillary, and inguinal regions), hepatosplenomegaly, scattered petechiae, and jaundice.

Laboratory investigations demonstrated hyperferritinemia (4,184 ng/mL), hepatic injury (ALT 111 U/L, AST 148 U/L, LDH 775 U/L), hypertriglyceridemia (2.70 mmol/L), elevated D-dimer (2.97 mg/L), and a marked hypercytokinemia pattern with elevated IL-6, IL-10, IFN-γ, and TNF-α (**Supplementary Table S3**). Hemoglobin was 101 g/L with mildly decreased platelets (161 × 10^9^/L). Lymphocyte subset analysis revealed decreased NK-cell percentage (8.25%) and absolute count (112.48 cells/μL; reference: 258–727), with reduced B cells and an inverted CD4/CD8 ratio (**Supplementary Table S3**). Immunoglobulins were polyclonally elevated (IgG 18.05 g/L, IgA 2.53 g/L, IgE >2,500 IU/mL). ANA was positive (granular, 1:80) but ENA, ANCA, and all other autoantibodies were negative. Mycoplasma pneumoniae IgM was weakly positive (±); quantitative PCR for EBV, HSV, TB, and MP in blood were negative. Blood cultures, bone marrow cultures, and brucella antibodies were all negative.

Bone marrow aspiration showed active proliferative marrow without hemophagocytosis. Cervical lymph node biopsy demonstrated reactive lymphoid hyperplasia with acute lymphadenitis; EBER *in situ* hybridization and acid-fast staining were negative; tNGS of lymph node tissue detected no pathogens (detailed immunohistochemistry in **Supplementary Material**).

Although the clinical picture was suggestive of HLH (hyperferritinemia, elevated LDH, hypertriglyceridemia, hypercytokinemia, declining blood counts), complete HLH-2004 criteria were not fulfilled—notably, no hemophagocytosis was identified, sCD25 was not measured, and fibrinogen was normal. Diagnostic challenges during this episode included the unavailability of sCD25 measurement and NK-cell degranulation assays at that time, which precluded definitive HLH diagnosis and delayed recognition of the underlying cytotoxic immune vulnerability. The patient was treated with hepatoprotective therapy (oral glucuronolactone and intravenous reduced glutathione), with liver function normalizing within approximately one week. She was discharged after 5 days with ferritin declining from 4,184 to 1,877 ng/mL. She received outpatient oral prednisolone tapered over one month, with subsequent normalization of ferritin.

### 2.2 Second episode — initial presentation (March 2024)

Seven months later, the patient developed recurrent high-grade fever (peak 40.2 °C) unresponsive to antipyretics and progressive paroxysmal cough over 8 days. She received intravenous azithromycin, ceftriaxone, and methylprednisolone at an outside hospital without improvement. She acutely developed hypoxemia (SpO_2_ 89–93%) with respiratory distress, prompting emergent transfer to Tianjin Children’s Hospital on March 30, 2024.

On admission, the patient was acutely ill with altered mental status (GCS 12), tachypnea (RR 40/min), and SpO_2_ 89–93% on room air. Examination revealed diminished left lung breath sounds, hepatosplenomegaly (liver 1 cm, spleen 2 cm below costal margins), and delayed capillary refill (3 seconds).

### 2.3 Diagnostic workup

Serial hematology revealed progressive pancytopenia: hemoglobin declined to a nadir of 72 g/L, platelets to 32 × 10^9^/L, and ANC to 0.86 × 10^9^/L, fulfilling the HLH-2004 cytopenia criterion in all three lineages. Coagulation studies demonstrated consumptive coagulopathy with fibrinogen nadir 0.748 g/L, D-dimer peak 20.08 mg/L, and prolonged PT and TT. Hepatic injury was severe (ALT 1,707 U/L, AST 4,017 U/L, LDH 6,464 U/L) with hypoalbuminemia (28.4 g/L) and elevated blood ammonia (117 μg/dL). Triglycerides peaked at 3.42 mmol/L. Serum ferritin peaked at 31,905 ng/mL (>200-fold ULN) and progressively declined to 278 ng/mL at discharge (serial trends in **Supplementary Table S3**).

Immune activation markers demonstrated sCD25 of 9,249 pg/mL and fulminant hypercytokinemia: IL-6 273.4 pg/mL, IL-10 1,765.0 pg/mL, IFN-γ 236.5 pg/mL, and TNF-α 28.7 pg/mL (**Supplementary Table S3**). Bone marrow aspiration (April 3) was performed primarily to exclude hematologic malignancy; flow cytometry excluded clonal lymphoid or myeloid populations and karyotype was normal (46, XX). Morphological examination additionally demonstrated hemophagocytosis—macrophages engulfing erythrocytes and nucleated cells—with impaired megakaryocyte platelet production. While hemophagocytosis can be observed in critical illness and sepsis and is not pathognomonic of HLH, it was interpreted as supportive evidence within the full clinical and laboratory context (2, 3, 18).

CSF analysis (April 3) showed mildly elevated protein (866 mg/L) with minimal pleocytosis (3 WBC/mm³); tNGS and culture were negative for all pathogens. Targeted NGS of sputum detected M. pneumoniae (1.74 × 10^4^ copies/mL, Category A), confirmed by BAL fluid tNGS (6.95 × 10^5^ copies/mL). Serum MP-IgM was 1:640 positive. EBV-DNA, EBV antibodies, and all other viral and bacterial workup were negative.

The patient fulfilled 7 of 8 HLH-2004 criteria: (1) fever >7 days; (2) splenomegaly; (3) trilineage cytopenia; (4) hypertriglyceridemia and hypofibrinogenemia; (5) bone marrow hemophagocytosis; (6) decreased NK-cell activity; (7) ferritin 31,905 ng/mL. Soluble CD25 was 9,249 pg/mL (approximately 2,312 U/mL using the standard conversion factor of ~4 pg/mL per U/mL), marginally below the HLH-2004 threshold of ≥2,400 U/mL but substantially above normal and indicative of marked T-cell activation. The HScore was 231 (>99% probability; **Supplementary Table S1**). While HLH-2004 criteria and the HScore were not designed as diagnostic standards for secondary HLH and have recognized limitations in this setting (3, 18), the combination of 7 fulfilled criteria (well exceeding the 5/8 diagnostic threshold), compatible clinical phenotype, borderline-elevated sCD25 confirming T-cell activation, and an identified trigger collectively supported a diagnosis of MP-triggered HLH.A comparison between episodes is provided in **Supplementary Table S3**.

### 2.4 Respiratory complications — plastic bronchitis

Chest CT on admission demonstrated extensive left lung consolidation with atelectasis, bilateral pleural effusions (left predominant), and scattered right lung infiltrates (**Figure 1A**). Pulmonary CTA excluded embolism. Given persistent hypoxemia and left lung non-expansion, emergent bronchoscopy (March 30) revealed left lower lobe obstruction by plastic cast-like mucus plugs, which were extracted with foreign body forceps (**Figure 1B**) with immediate ventilatory improvement.

**Figure 1:**
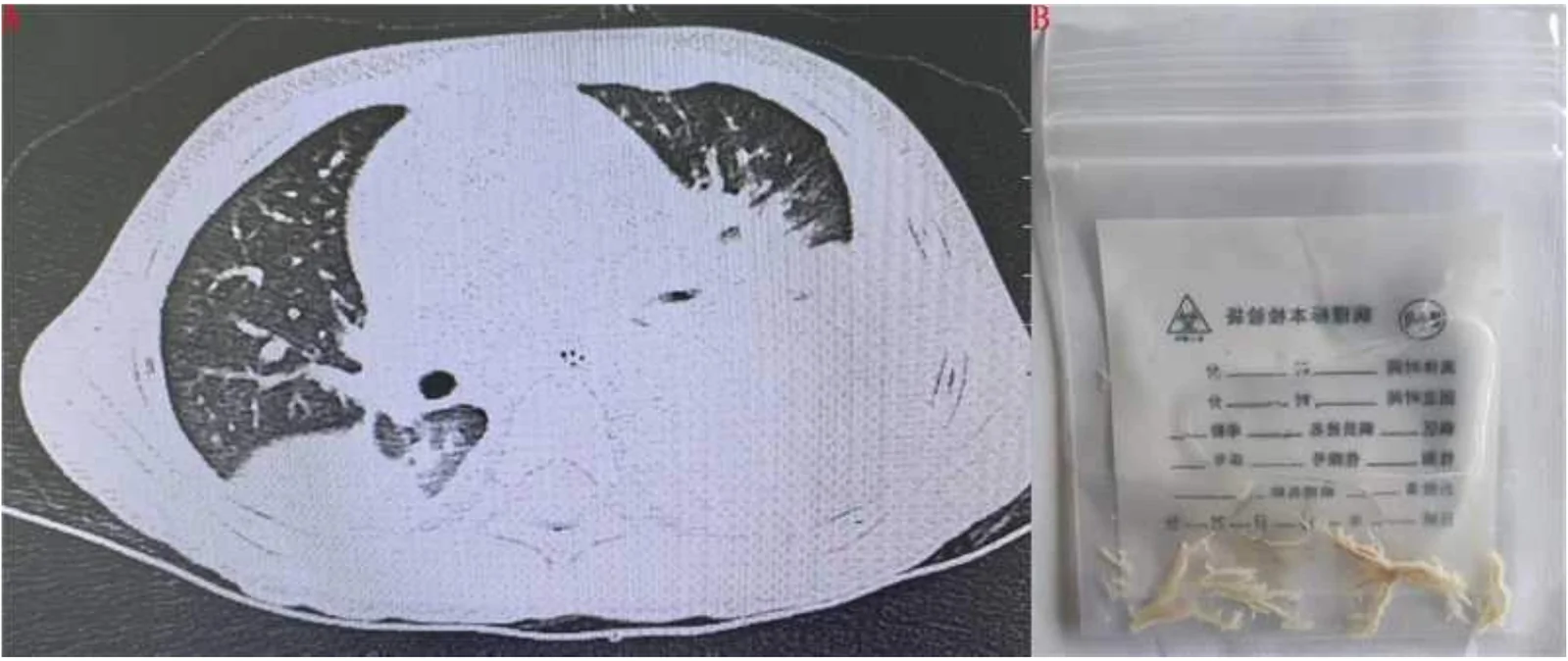
Imaging and bronchoscopy findings. **(A)** Chest CT showing extensive left lung consolidation with atelectasis and bilateral pleural effusions (left predominant). **(B)** Bronchial cast retrieved via flexible bronchoscopy, exhibiting a tree-like branching morphology.

A second bronchoscopy (April 5) identified recurrent mucofibrinous casts obstructing both left lower lobe and right lower lobe bronchi; bilateral cast extraction again improved ventilation. Histopathological examination confirmed type I (inflammatory) plastic bronchitis, with immunohistochemistry demonstrating CD3(+), CD68(+), and MPO(+) infiltrate without fungal elements.

### 2.5 Neurological complication

On hospital day 3, the patient experienced a single generalized tonic-clonic seizure lasting 90 seconds, which terminated spontaneously. Head CT excluded hemorrhage. Brain MRI demonstrated bilateral parietal white matter T2/FLAIR hyperintensities and mild cerebellar swelling, attributed to toxic–metabolic encephalopathy in the context of cytokine storm and hepatic dysfunction (ammonia 117 μg/dL). No further seizures occurred.

### 2.6 Immunological evaluation

Comprehensive immunological screening was performed to identify HLH predisposition (**Supplementary Table S2**). NK-cell degranulation (CD107a assay) yielded ΔCD107a of 5.68%, within the borderline zone (≤5% = severely impaired; 5–10% = possible impairment). CTL degranulation was normal (ΔMFI 5.4; threshold ≥2.8). Flow cytometric protein expression screening demonstrated normal levels of perforin, MUNC13-4, granzyme B, SAP, and XIAP in both NK cells and CTLs, making classical XLP1/XLP2 and familial HLH types 3–5 unlikely at the protein-screening level, though not definitively excluded (detailed values in **Supplementary Table S2**). Notably, NK-cell perforin expression (84.62%) was only marginally above the lower threshold (≥81%). Whole-exome sequencing was not performed during this hospitalization due to the critical clinical status and logistical constraints; high-coverage (≥200×) WES or whole-genome sequencing (WGS) was planned for outpatient follow-up after clinical stabilization.

NK-cell counts were low during the first episode (112.48 cells/μL; reference 258–727); however, quantitative NK-cell deficiency is a common finding in pediatric critical illness and systemic inflammation (22), and this finding cannot be confidently attributed to a baseline deficiency without confirmation during quiescence. Notably, comprehensive T-cell functional assessment—including proliferation assays, Th17 differentiation capacity, and IL-17/IL-22 production—was not performed during this hospitalization but was recommended for outpatient evaluation, given that defective Th17 responses may predispose to severe infections with cellular organisms such as *M. pneumoniae.*

### 2.7 Treatment and outcome

Treatment was initiated with a modified HLH-2004 protocol: intravenous dexamethasone 10 mg/m²/day and IVIG 500 mg/kg over 2 days; etoposide was withheld given the infectious etiology and favorable response. Dexamethasone was subsequently transitioned to hydrocortisone due to drug hypersensitivity, then tapered to oral prednisolone before discharge. Antimicrobial therapy included meropenem for broad-spectrum coverage and oral doxycycline targeting M. pneumoniae, with fluconazole added for antifungal prophylaxis. Supportive care comprised fresh frozen plasma and albumin infusions, supplemental oxygen, nasogastric nutritional support, hepatoprotective therapy, and electrolyte correction (detailed regimens in **Supplementary Material**).

Clinical response was evident within the first week. Fever resolved by day 8, oxygen was weaned by day 12, and respiratory status improved progressively after the second bronchoscopy. The patient was discharged on April 16 (day 17) in stable condition with resolving pancytopenia and declining liver enzymes. The clinical timeline is summarized in **Figure 2**.

**Figure 2:**
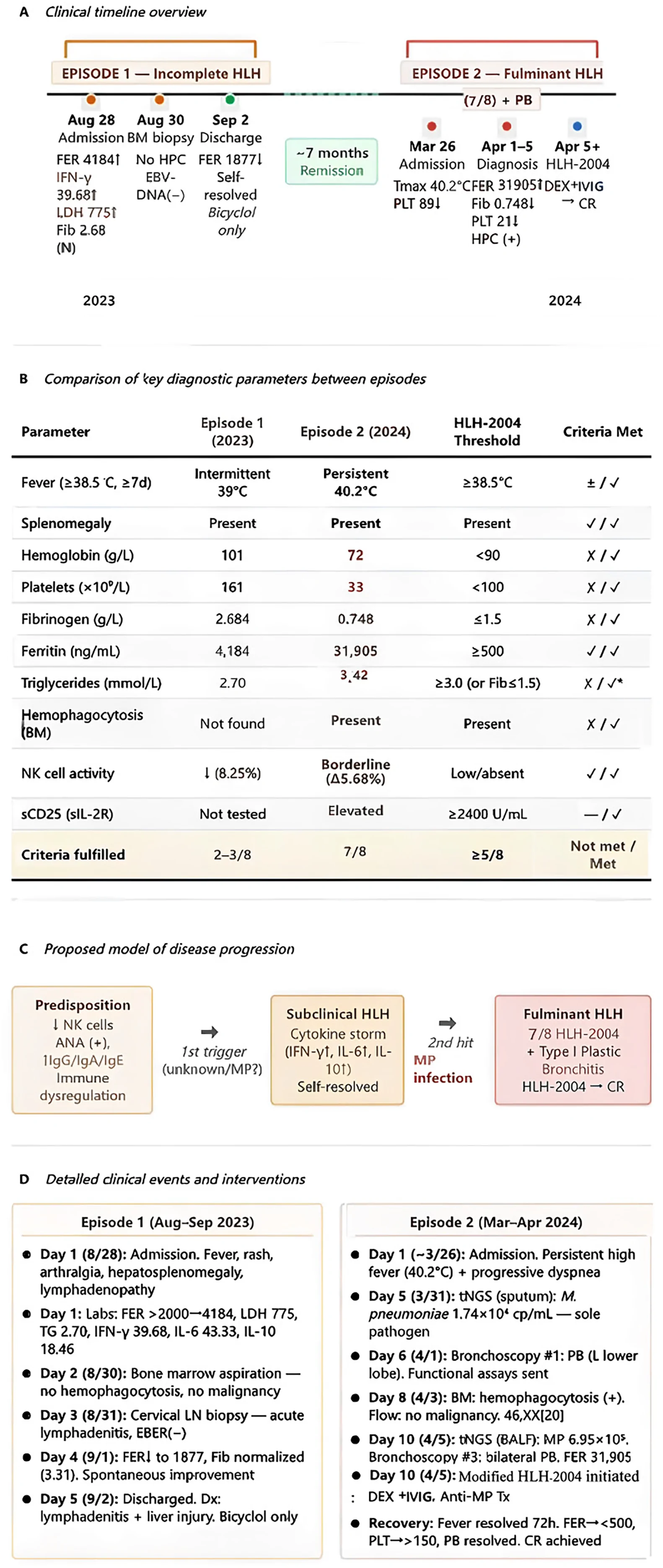
Clinical timeline of recurrent hemophagocytic lymphohistiocytosis (HLH) episodes. **(A)** Timeline overview showing two hyperinflammatory episodes separated by a 7-month remission. **(B)** Comparison of HLH-2004 diagnostic criteria between episodes: Episode 1 met 2–3/8 criteria (incomplete HLH, self-resolved), while Episode 2 fulfilled 7/8 criteria with borderline sCD25 (fulminant HLH, requiring HLH-2004 protocol). **(C)** Proposed “two-hit” pathogenesis model. Pre-existing immune dysregulation (↓NK cells, ANA+, elevated immunoglobulins) served as the predisposing background; Mycoplasma pneumoniae infection acted as the second hit, triggering fulminant HLH with concurrent type I plastic bronchitis. **(D)** Detailed clinical events and interventions for each episode.

### 2.8 Follow-up

Complete clinical and biochemical recovery was confirmed at outpatient follow-up, with normalization of all inflammatory markers, ferritin, liver function, and blood counts. Oral prednisolone was tapered over approximately 4 weeks and then discontinued; doxycycline was completed without adverse effects. No long-term immunomodulatory therapy (e.g., cyclosporine) was initiated, as the patient achieved rapid and sustained remission following elimination of the infectious trigger, consistent with current recommendations that maintenance immunosuppression is not routinely required for infection-triggered secondary HLH responding to initial therapy (2, 18). Subsequent telephone follow-up confirmed continued well-being without recurrence of fever, cytopenias, or other symptoms. The family was counseled to seek immediate medical evaluation for any febrile illness. Long-term immunological surveillance was recommended given the borderline NK-cell degranulation and recurrent hyperinflammatory history. High-coverage (igh-co whole-exome or whole-genome sequencing targeting immune dysregulation genes was strongly recommended to identify potential hypomorphic or novel variants undetectable by conventional panels. Family history was notable for the patient’s mother and maternal grandmother carrying diagnoses of “rheumatoid arthritis-like joint disease,” raising the possibility of inherited immune dysregulation predisposition.

## 3 Discussion

This case illustrates the convergence of infection-triggered hyperinflammation, severe pulmonary disease, and a possible host cytotoxic-immune vulnerability. The patient experienced two hyperinflammatory episodes separated by 7 months; only the second had microbiological evidence establishing *M. pneumoniae* as the trigger. We therefore characterize this as recurrent hyperinflammation rather than recurrent MP-HLH, as the first episode neither fulfilled HLH-2004 criteria nor had a confirmed pathogen. That episode met 7 of 8 HLH-2004 criteria (2), with sCD25 marginally below threshold but confirming T-cell activation—used here as supportive rather than definitive diagnostic evidence—and was further complicated by severe hepatic injury, consumptive coagulopathy, toxic–metabolic encephalopathy, polyserositis, and histologically confirmed type I PB. The prior HLH-like episode, reduced NK-cell number, and borderline NK-cell degranulation suggest that the conventional primary/secondary HLH distinction is insufficient in this child; instead, the findings support a threshold model in which infectious challenge interacts with incompletely characterized host susceptibility to produce uncontrolled inflammation (1–3).

MP-HLH is not invariably mild. While earlier reports described moderate cytopenias responsive to corticosteroids or IVIG alone (5, 12, 13), Lu et al. documented rapid progression with respiratory failure and one death (14), and Yang et al. found higher mortality when diagnosis was delayed beyond 2 weeks (7). Compared with most previously reported survivors, the present patient exhibited unusually severe hyperferritinemia, hepatic injury, coagulopathy, neurological involvement, and bilateral recurrent bronchial casts, reinforcing that persistent fever with unexplained cytopenia or coagulopathy during refractory MP pneumonia should prompt immediate HLH evaluation. The cytokine elevations—particularly IL-10 (1,765 pg/mL)—are consistent with macrophage activation, though they are non-specific and only moderately elevated compared with published severe HLH cohorts; they should be interpreted as supportive of the clinical diagnosis rather than confirmatory of a specific immunopathogenic mechanism (6).

The favorable response to corticosteroids and IVIG without etoposide raises the question of whether this presentation represents MAS or hyperferritinemic sepsis (HFS) rather than classical HLH. These entities share hyperferritinemia, cytopenias, coagulopathy, and multiorgan dysfunction, and current diagnostic frameworks cannot reliably distinguish them without confirmed genetic cytotoxicity defects (3, 18, 20). MAS exists on a continuum with secondary HLH, particularly when infection-triggered in a host with immune dysregulation (20). In our patient, fulfillment of 7/8 HLH-2004 criteria, bone marrow hemophagocytosis, borderline NK-cell degranulation, and an identified trigger support a diagnosis within the HLH/MAS spectrum, but definitive nosological distinction requires genetic confirmation. IL-18—characteristically elevated in MAS and useful for differentiation (21)—was not measured, representing a diagnostic limitation. Regardless of precise classification, the clinical imperative—prompt immunosuppression combined with trigger elimination—remains identical across these overlapping syndromes (18, 20).

The pulmonary findings provide a potential mechanistic link between localized airway inflammation and systemic HLH (**Figure 3**). The CD3-, CD68-, and MPO-positive fibrinous casts confirmed type I inflammatory PB under the Seear classification (8). M. pneumoniae damages ciliated epithelium, impairs mucociliary clearance, and induces intense neutrophilic inflammation favoring cast formation (9), while concurrent coagulation–fibrinolysis activation is well documented in pediatric PB cohorts (15). We hypothesize that cytokine-mediated epithelial barrier injury permitted leakage of fibrinogen-rich plasma into the airway where local tissue-factor activity converted it to fibrin, while simultaneous systemic consumption produced the low circulating fibrinogen. The extreme D-dimer elevation supports this parallel local and systemic coagulation response. Bronchial casts were noted in two of three children reported by Lu et al. (14); however, pathological confirmation as type I PB in the setting of HLH has not been previously documented. We acknowledge that the proposed mechanistic link between HLH-associated coagulopathy and airway cast formation remains speculative; the primary clinical significance of this finding lies in alerting clinicians to the possibility of concurrent airway obstruction requiring emergent bronchoscopy during MP-HLH.

**Figure 3:**
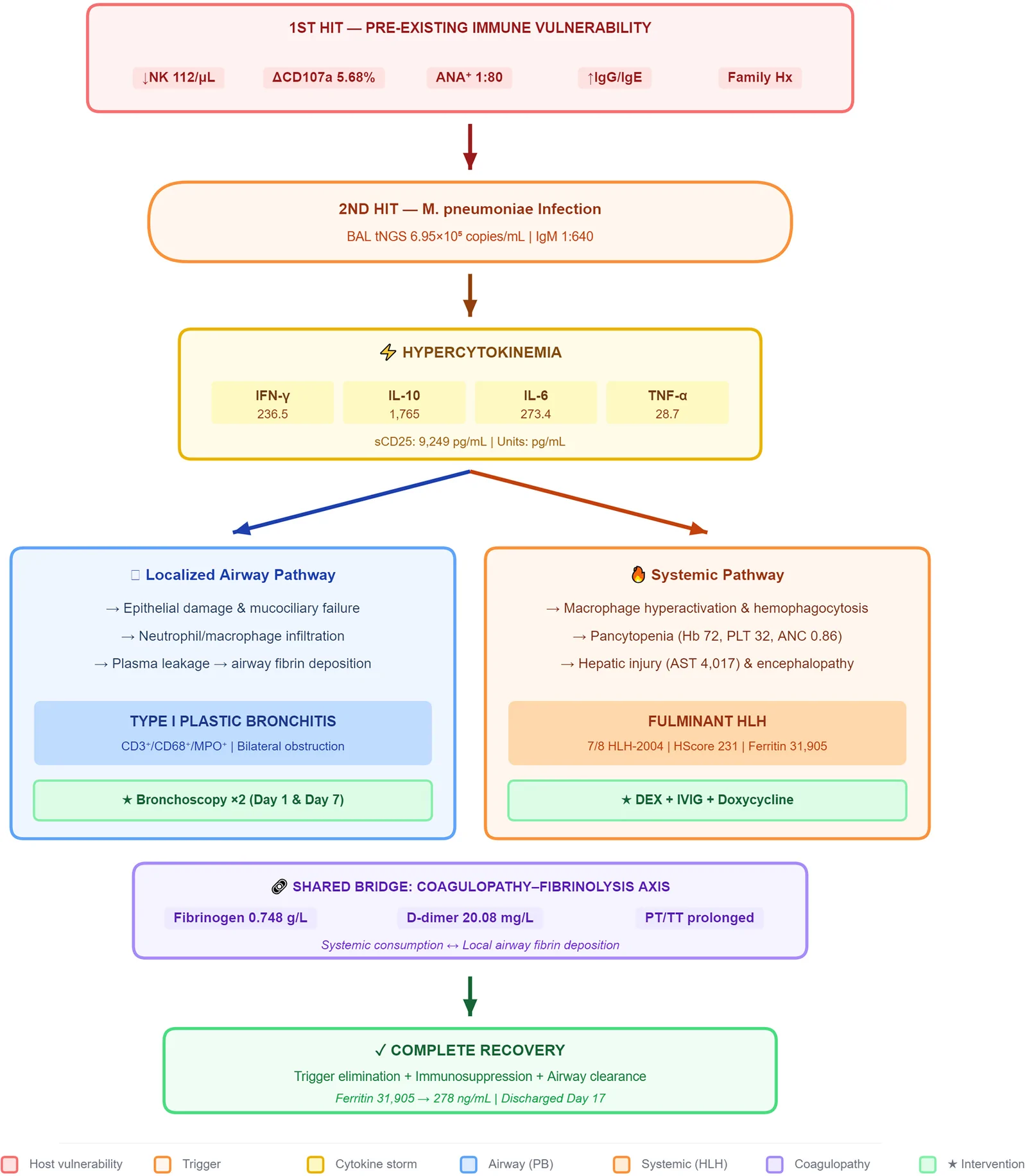
Proposed immunopathogenic model of concurrent type I plastic bronchitis and HLH. Pre-existing immune vulnerability (↓NK cells, borderline CD107a degranulation) constitutes the “first hit.” M. pneumoniae infection acts as the “second hit,” triggering hypercytokinemia that bifurcates into a localized airway pathway (neutrophil infiltration, fibrin deposition → type I PB) and a systemic pathway (macrophage hyperactivation → fulminant HLH). The coagulopathy–fibrinolysis axis bridges both processes. Patient-specific values are shown; stars (★) indicate intervention points.

The borderline NK-cell degranulation (ΔCD107a 5.68%) is noteworthy but requires cautious interpretation, particularly as it was measured during active disease when inflammation-mediated suppression may depress NK-cell function independently of intrinsic defects. Defective cytotoxic-granule exocytosis prolongs immune-synapse contact, sustains antigen presentation, and amplifies IFN-γ–driven macrophage activation (16). CD107a mobilization screens for degranulation disorders involving UNC13D, STX11, STXBP2, RAB27A, LYST, and AP3B1 (10); values below 5% carry 96% sensitivity for genetic defects, whereas values of 5–10% require integration with CTL assays, protein expression, and molecular data (11). Perforin and CD107a assays discriminate genetic cytotoxicity defects more effectively than conventional NK killing assays (17). In this patient, CTL degranulation was normal and perforin, MUNC13-4, SAP, and XIAP expression was preserved, reducing the likelihood of classical familial HLH but not excluding hypomorphic variants, functional mutations preserving protein quantity, or acquired inflammation-related suppression. Since both the borderline degranulation and quantitative NK-cell deficiency were documented during active inflammatory episodes—and reduced NK-cell numbers are well recognized in pediatric critical illness (22)—reassessment during confirmed remission is essential to distinguish intrinsic from acquired dysfunction (10, 11, 17, 18).

Several clinical implications emerge. First, HLH should be actively considered during macrolide-refractory MP pneumonia accompanied by persistent fever, progressive cytopenia, falling fibrinogen, or rising ferritin; treatment should not be delayed pending genetic results (2, 18). Second, abrupt lobar collapse or refractory hypoxemia warrants early bronchoscopy, as casts may recur bilaterally. Third, recurrent or unusually severe infection-triggered hyperinflammation warrants functional immune screening—including CD107a degranulation, perforin expression, SAP/XIAP assessment, and T-cell functional evaluation (proliferation, Th17 pathway)—and high-coverage (≥200×) WES/WGS for definitive genetic characterization, even in previously healthy children (10, 18, 19).

This report has several strengths: histopathological confirmation of type I PB in the setting of HLH, comprehensive functional cytotoxic screening including CD107a degranulation and protein expression panels, and longitudinal documentation of two hyperinflammatory episodes enabling comparison of disease evolution and host immune status. However, limitations must be acknowledged. It describes a single patient; *M. pneumoniae* was not confirmed during the first episode, and that episode did not meet full HLH-2004 criteria, precluding the designation of recurrent MP-HLH; rather, the pattern represents recurrent hyperinflammation of which only the second was definitively MP-triggered HLH. Importantly, two key investigations were not completed prior to manuscript submission and represent significant limitations (1): high-coverage (igh-covera WES/WGS, which is essential for identifying underlying immune dysregulation and hypomorphic variants in patients with recurrent severe HLH (19), was not performed due to the acute clinical status and remains planned for outpatient follow-up; and (2) comprehensive T-cell functional assessmentivesionngy proliferation assays and Th17 pathway evaluation (IL-17/IL-22 production, Th17 differentiation capacity)iati not conducted, precluding characterization of potential adaptive immune defects predisposing to severe cellular organism infections. Additionally, CD107a degranulation was measured only once during active disease, and reassessment during confirmed remission is needed to distinguish intrinsic from inflammation-related suppression. The proposed relationship between systemic hypofibrinogenemia, local airway fibrin deposition, and PB remains mechanistic inference requiring validation.

## 4 Conclusion

This case demonstrates that MP-associated HLH can present with fulminant multiorgan failure and type I plastic bronchitis in previously healthy children, underscoring the need for early recognition, prompt HLH-directed therapy, and emergent bronchoscopy. Borderline NK-cell degranulation combined with recurrent hyperinflammation supports a continuum between primary and secondary HLH, warranting comprehensive functional cytotoxic screening and high-coverage genomic sequencing to guide long-term management.

## 5 Patient perspective

The patient’s parents reported: “Our daughter’s rapid deterioration and seizures were terrifying. The bronchoscopies brought immediate breathing relief. After discharge, she returned to school and normal life. We strictly follow all follow-up appointments and remain vigilant for febrile episodes given her immune vulnerability.

## Data Availability

The raw data supporting the conclusions of this article will be made available by the authors, without undue reservation.

## Glossary

ADA: Adenosine deaminase
ALT: Alanine aminotransferase
ANA: Antinuclear antibody
ANC: Absolute neutrophil count
ANCA: Anti-neutrophil cytoplasmic antibody
APTT: Activated partial thromboplastin time
ASO: Anti-streptolysin O
AST: Aspartate aminotransferase
BALF: Bronchoalveolar lavage fluid
BM: Bone marrow
cANCA: Cytoplasmic anti-neutrophil cytoplasmic antibody
CR: Complete remission
CRP: C-reactive protein
CSF: Cerebrospinal fluid
CT: Computed tomography
CTA: Computed tomography angiography
CTL: Cytotoxic T lymphocyte
DC: Dendritic cell
DEX: Dexamethasone
DIC: Disseminated intravascular coagulation
DNA: Deoxyribonucleic acid
EBER: Epstein–Barr virus-encoded small RNA
EBV: Epstein–Barr virus
EEG: Electroencephalography
ENA: Extractable nuclear antigen
ESR: Erythrocyte sedimentation rate
FDC: Follicular dendritic cell
Fg: Fibrinogen
FHL: Familial hemophagocytic lymphohistiocytosis
FLAIR: Fluid-attenuated inversion recovery
GCS: Glasgow Coma Scale
HIT: Heparin-induced thrombocytopenia
HLA: Human leukocyte antigen
HLH: Hemophagocytic lymphohistiocytosis
HSV: Herpes simplex virus
IFN-γ: Interferon-gamma
IgA: Immunoglobulin A
IgE: Immunoglobulin E
IgG: Immunoglobulin G
IgM: Immunoglobulin M
IL: Interleukin
INR: International normalized ratio
IVIG: Intravenous immunoglobulin
JAK-STAT: Janus kinase–signal transducer and activator of transcription
LAMP-1: Lysosomal-associated membrane protein 1
LDH: Lactate dehydrogenase
MAS: Macrophage activation syndrome
MCP-1: Monocyte chemoattractant protein-1
MFI: Mean fluorescence intensity
MP: Mycoplasma pneumoniae
MP-HLH: Mycoplasma pneumoniae-associated hemophagocytic lymphohistiocytosis
MPO: Myeloperoxidase
MRI: Magnetic resonance imaging
MUC5AC: Mucin 5AC
NF-κB: Nuclear factor kappa-light-chain-enhancer of activated B cells
NK: Natural killer
pANCA: Perinuclear anti-neutrophil cytoplasmic antibody
PAI-1: Plasminogen activator inhibitor-1
PB: Plastic bronchitis
PCR: Polymerase chain reaction
PCT: Procalcitonin
PF4: Platelet factor 4
PT: Prothrombin time
QFT: QuantiFERON (tuberculosis interferon-γ release assay)
RF: Rheumatoid factor
SAP: Signaling lymphocytic activation molecule-associated protein
sCD25: Soluble CD25
sIL-2R: Soluble interleukin-2 receptor
T2WI: T2-weighted imaging
TB: Tuberculosis
TF: Tissue factor
TG: Triglycerides
TLR: Toll-like receptor
tNGS: Targeted next-generation sequencing
TNF-α: Tumor necrosis factor-alpha
TT: Thrombin time
ULN: Upper limit of normal
WBC: White blood cell
WES: Whole-exome sequencing
XIAP: X-linked inhibitor of apoptosis protein
XLP: X-linked lymphoproliferative disease
γ-GT: Gamma-glutamyl transferase
