# Supplementary material for: Mycoplasma pneumoniae-triggered hemophagocytic lymphohistiocytosis complicated by type I plastic bronchitis in a child: a case report
Source: Front Immunol. 2026 Oct 29;17:1944516. doi: 10.3389/fimmu.2026.1944516 (PMC13756142; doi:10.3389/fimmu.2026.1944516)
Supplement: Supplementary file 3 [file Table1.pdf]

Supplementary Table S1: HScore Calculation

HScore Component	Patient Value	Points
Known underlying immunosuppression	No	0
Temperature	40.2°C (>39.4°C)	49
Organomegaly	Hepatosplenomegaly	38
Number of cytopenias	3 lineages	34
Ferritin	31,905 ng/mL (>6,000)	50
Triglycerides	3.42 mmol/L (≥3.0)	44
Fibrinogen	0.748 g/L (≤2.5)	30
AST	4,017 U/L (≥30)	19
Hemophagocytosis on bone marrow aspirate	Present	35
**Total HScore**	**Probability of HLH >99%**	**231**

Exclusionary Workup

Investigation	Result	Conclusion
Bone marrow flow cytometry (immunophenotyping)	No aberrant immunophenotype; CD34+ cells 0.1%	Hematological malignancy excluded
Chromosome karyotype (G-banding, 20 metaphases)	46,XX[20]	Normal female; clonal cytogenetic abnormality excluded
NK functional protein panel (Perforin, Granzyme B, MUNC13-4, SAP, XIAP)	All normally expressed	Classical primary HLH (FHL2-5, XLP1/2) excluded
Anti-PF4/heparin IgG antibody (HIT test)	Negative	Heparin-induced thrombocytopenia excluded
CSF tNGS (bacteria, fungi, viruses, atypical pathogens)	All negative	CNS infection excluded

Final Diagnosis: Secondary hemophagocytic lymphohistiocytosis triggered by Mycoplasma pneumoniae infection, complicated by Type I inflammatory plastic bronchitis (pathologically confirmed), multi-organ dysfunction (hepatic failure, disseminated intravascular coagulation, respiratory failure requiring mechanical ventilation, metabolic encephalopathy with seizures), and polyserositis (bilateral pleural effusion, pericardial effusion, pelvic effusion).
