# Supplementary material for: Mycoplasma pneumoniae-triggered hemophagocytic lymphohistiocytosis complicated by type I plastic bronchitis in a child: a case report
Source: Front Immunol. 2026 Oct 29;17:1944516. doi: 10.3389/fimmu.2026.1944516 (PMC13756142; doi:10.3389/fimmu.2026.1944516)
Supplement: Supplementary file 4 [file Table2.pdf]

Supplementary Table S2. NK Cell Functional Protein Screening and Degranulation Assay Results

Protein / Assay	Associated Gene	Associated Disease	NK Cells (%)	Reference	CTL (%)	Reference	Interpretation
Perforin	PRF1	FHL2	84.62	≥81%	68.32	≥2%	Normal
Granzyme B	—	—	84.09	≥77%	66.39	≥6%	Normal
MUNC13-4	UNC13D	FHL3	63.16	≥51%	77.31	≥60%	Normal
SAP (SH2D1A)	SH2D1A	XLP1	99.25	≥26%	93.82	≥43%	Normal
XIAP	XIAP/BIRC4	XLP2	65.93	≥59%	77.46	≥61%	Normal
**CD107a degranulation**	UNC13D, STX11, STXBP2	FHL3/4/5	**Δ5.68%**	≥5–10%	ΔMFI 5.4	≥2.8	**Borderline low**

Summary	Findings
Classical FHL protein deficiency	**Excluded** — all 5 proteins normally expressed in both NK and CTL compartments
Functional NK degranulation capacity	**Borderline impaired** — ΔCD107a at lower limit of normal range (5.68%; threshold 5–10%)
Clinical significance	NK cells are structurally equipped (normal granule content) but may exhibit suboptimal cytotoxic degranulation function, representing a potential immunological susceptibility factor for infection-triggered secondary HLH in this patient

Note: The CD107a degranulation assay measures upregulation of LAMP-1 on NK/CTL cell surfaces upon K562 target cell stimulation, reflecting the functional capacity of cytotoxic granules to fuse with the plasma membrane.
