# Supplementary material for: Mycoplasma pneumoniae-triggered hemophagocytic lymphohistiocytosis complicated by type I plastic bronchitis in a child: a case report
Source: Front Immunol. 2026 Oct 29;17:1944516. doi: 10.3389/fimmu.2026.1944516 (PMC13756142; doi:10.3389/fimmu.2026.1944516)
Supplement: Supplementary file 5 [file Table3.pdf]

Supplementary Table S3 Comparison of Clinical Features and Laboratory Findings Between Two Episodes of Cytokine Storm

Parameter	Episode 1 (Aug 2023)	Episode 2 (Mar–Apr 2024)	Reference Range
**Demographics**			
Age at onset	6 years	6 years 7 months	—
Duration of hospitalization	5 days	17 days	—
Identified trigger	Undetermined	Mycoplasma pneumoniae	—
**Clinical Manifestations**			
Fever (≥38.5°C)	+	+	—
Hepatosplenomegaly	Hepatomegaly	Hepatosplenomegaly	—
Skin rash	+	+	—
Lymphadenopathy	+ (cervical)	+	—
Respiratory failure	−	**+ (mechanical ventilation)**	—
Seizures	−	+	—
Serous effusion	−	**+ (pleural, pericardial, pelvic)**	—
Plastic bronchitis	−	**+ (Type I, pathologically confirmed)**	—
**Hematology**			
WBC (×10⁹/L)	12.56	4.23	4.0–12.0
Hemoglobin (g/L)	108	**72**	110–155
Platelets (×10⁹/L)	134	**33 (nadir)**	100–400
Neutrophils (×10⁹/L)	8.74	**0.86**	1.5–8.0
**Inflammatory / HLH Markers**			
Ferritin (ng/mL)	**4,184**	**31,905 (peak)**	10–120
sCD25 / sIL-2R (pg/mL)	NT	**9,249**	<2,400
IL-6 (pg/mL)	**87.6**	**273.4**	<7
IL-10 (pg/mL)	**495.2**	**1,765.0**	<9.1
IFN-γ (pg/mL)	**126.8**	**236.5**	<18
TNF-α (pg/mL)	15.3	**28.7**	<23
NK cell proportion (%)^[a]	**2.76**	**3.12**	7–40
**Hepatic Function**			
ALT (U/L)	**286**	**1,707**	0–40
AST (U/L)	**342**	**4,017**	0–40
LDH (U/L)	**856**	**6,464**	120–300
Total bilirubin (μmol/L)	18.6	**52.3**	0–21
Albumin (g/L)	**32.4**	**22.8**	35–55
**Coagulation**			
Fibrinogen (g/L)	2.14	**0.748**	2.0–4.0

Parameter	Episode 1 (Aug 2023)	Episode 2 (Mar–Apr 2024)	Reference Range
D-dimer (mg/L)	**1.86**	**20.08**	<0.5
PT (s)	13.2	**18.6**	11–14
APTT (s)	34.5	**52.3**	25–37
**Lipid Metabolism**			
Triglycerides (mmol/L)	**2.18**	**3.42**	<1.7
**Bone Marrow**			
Hemophagocytosis	Not performed	**Confirmed (10/16 macrophages with phagocytosis)**	—
**Pathogen Detection (tNGS)**			
BALF	Not performed	M. pneumoniae 6.95×10^5 copies/mL	—
Sputum	Not performed	M. pneumoniae 1.74×10^4 copies/mL	—
CSF	Not performed	Negative (all panels)	—
**Diagnosis & Outcome**			
Diagnosis	Subclinical MAS / Incomplete HLH	**Fulminant HLH (8/8 criteria met)**	—
HScore	—	**231 points (>99% probability)**	>169
Treatment	Hepatoprotection only	HLH-2004 (DEX + IVIG) + antibiotics	—
Outcome	Spontaneous resolution	Recovery after immunosuppression	—

^a NK cell proportion is expressed as a percentage of total leukocytes. During Episode 1, lymphocyte subset analysis reported NK cells (CD16+CD56+) as 8.25% of lymphocytes (absolute count 112.48 cells/μL), corresponding to 2.76% of total leukocytes; during Episode 2, NK cells constituted 3.12% of total leukocytes.
